# Black Raspberry (*Rubus* *coreanus* Miquel) Promotes Browning of Preadipocytes and Inguinal White Adipose Tissue in Cold-Induced Mice

**DOI:** 10.3390/nu11092164

**Published:** 2019-09-10

**Authors:** Woo Yong Park, Seong-Kyu Choe, Jinbong Park, Jae-Young Um

**Affiliations:** 1 Department of Science in Korean Medicine, Graduate School, Kyung Hee University, 26 Kyungheedae-ro, Dongdaemun-Gu, Seoul 02447, Korea; 2 Department of Pharmacology, College of Korean Medicine, Kyung Hee University, Seoul 02447, Korea; 3 Basic Research Laboratory for Comorbidity Regulation, Comorbidity Research Institute, Kyung Hee University, Seoul 02447, Korea; 4 Department of Microbiology, Wonkwang University School of Medicine, Iksan 54538, Korea

**Keywords:** black raspberry, ellagic acid, obesity, beige adipocyte (fat), uncoupling protein 1, thermogenesis

## Abstract

The alteration of white adipose tissue (WAT) “browning”, a change of white into beige fat, has been considered as a new therapeutic strategy to treat obesity. In this study, we investigated the browning effect of black raspberry (*Rubus coreanus* Miquel) using in vitro and in vivo models. Black raspberry water extract (BRWE) treatment inhibited lipid accumulation in human mesenchymal stem cells (hMSCs) and zebrafish. To evaluate the thermogenic activity, BRWE was orally administered for 2 weeks, and then, the mice were placed in a 4 °C environment. As a result, BRWE treatment increased rectal temperature and inguinal WAT (iWAT) thermogenesis by inducing the expression of beige fat specific markers such as PR domain zinc-finger protein 16 (PRDM16), uncoupling protein 1 (UCP1), peroxisome proliferator-activated receptor gamma coactivator 1-alpha (PGC1α), and t-box protein 1 (TBX1) in cold-exposed mice. Furthermore, ellagic acid (EA), a constituent of BRWE, markedly promoted beige specific markers: UCP1, PGC1α, TBX1, and nuclear respiratory factor 1 in beige differentiation media (DM)-induced 3T3-L1 adipocytes. Our findings indicate that BRWE can promote beige differentiation/activation, and EA is the active compound responsible for such effect. Thus, we suggest the nature-derived agents BRWE and EA as potential agents for obesity treatment.

## 1. Introduction

Obesity, associated with a caloric imbalance in the body, has become an international public health problem worldwide [1]. The etiological relationship between obesity and metabolic diseases has been established by various experimental and clinical studies [2]. Briefly, the massive expansion of white adipose tissue (WAT), a striking feature in obesity, resulted in an increased risk of chronic disorders including type 2 diabetes, heart diseases, systemic hypertension, hyperlipidemia, and arteriosclerosis [3].

Human adipose tissue can be divided into two subsets: brown adipose tissue (BAT) and WAT. The WAT is used for energy storage while the BAT is involved in energy dissipation through heat generation from free fatty acids (FFAs) [4]. Thermogenic ability of BAT depends on a large number of mitochondria and a high level of uncoupling protein 1 (UCP1). The UCP1 has been known as one of the key factors in energy homeostasis and mediates the adaptive thermogenesis in adipose tissues [5]. Interestingly, it has recently been shown that the WAT contributes to thermogenesis via various stimulants including cold exposure and β3 adrenergic receptor (β3 AR) agonist [6]. This physiological phenomenon is called “browning”, and this type of WAT is named “beige adipocyte” [7]. The beige adipocytes promote energy expenditure by activating UCP1 expression as compared with the common WAT [8]. Therefore, the pharmacological alteration of WAT function white change into beige fat has been considered new therapeutic strategies to prevent and treat obesity [9].

Black raspberry (BR), which belongs to genus *Rubus* (Rosaceae), is one of the traditional medicines used for the treatment of impotence, spermatorrhea, enuresis, and asthma in Northeast Asia [10]. Recent studies have shown the pharmacological properties of BR includes anticarcinogenic, antioxidant, and anti-inflammation [11]. Our previous study demonstrated that BR has an anti-obesity effect through brown adipose tissue (BAT) activation in obese mice [12]. However, the anti-obesity mechanism of BR regarding browning of WAT is not fully elucidated. The present study clarified the anti-obesity mechanism of BR in beige-differentiated adipocytes and in mice undergoing a cold-stress test. Moreover, the study investigated whether BR-derived phytochemicals can induce beige differentiation.

## 2. Materials and Methods

### 2.1. Reagents

Dulbecco modified eagle medium (DMEM) medium and fetal bovine serum (FBS) were obtained from Gibco (Grand Island, NY, USA). Insulin, 3-isobutylmethylxanthine (IBMX), indomethacin, 3,3′,5-triiodo-L-thyronine (T3), dexamethasone, troglitazone, Oil-red O powder, and CL316,243 were obtained from Sigma Aldrich (St Louis, MO, USA). Poly-vinylidene difluoride (PVDF) was procured from Millipore (Merck KGaA, Darmstadt, Germany). The electrochemiluminescence (ECL) kit was obtained from GE Healthcare Life Sciences (Seoul, Korea).

### 2.2. Sample Preparation

Dried BR was provided by Kyung Hee University Korean Hospital (Seoul, Korea). BR water extract (BRWE) was obtained by extracting BR in hot water at 100 °C for 3 h, followed by filtering (No 4, Whatman, Kent, UK). After being freeze-dried in a vacuum, it was dissolved in DMSO (20 mg·mL^−1^).

### 2.3. Cell Culture and Beige Adipocyte Differentiation

The human mesenchymal stem cells (hMSCs) were purchased from the Cell Engineering for Origin (Seoul, Korea). The cells were cultured in DMEM media supplemented with 10% FBS and 100 U mL^−1^ of penicillin and streptomycin in a CO_2_ incubator at 37 °C with 5% CO_2_ until confluence. The cells, on two days after confluence, were differentiated with differentiation medium (0.5 mM IBMX, 1 µM dexamethasone, 1 µg·mL^−1^ insulin, and 100 nM indomethacin) that was added to DMEM containing FBS 10% for two days (Days 2). Then, the medium was replaced by maintenance medium containing 1 µg·mL^−1^ insulin, 50 nM T3, and 0.5 µM troglitazone once every two days (three times, Days 2–6). On Day 2, BRWE was prepared in a maintenance medium at concentration of 5 and 10 µg mL^−1^.

3T3-L1 cells, a mouse embryo fibroblast cell line, were obtained from the American Type Culture Collection (Rockville, MD, USA). The cells were cultured in DMEM media supplemented with 10% FBS and 100 U mL^−1^ of penicillin and streptomycin in a CO_2_ incubator at 37 °C with 5% CO_2_ until confluence. The cells, two days after confluence, were differentiated with differentiation medium (0.5 mM IBMX, 0.5 µM dexamethasone, 1 µg·mL^−1^ insulin, 50 nM T3, and 0.5 µM troglitazone) and were added to DMEM containing FBS 10% for two days (Days 2). Then, the medium was replaced by maintenance medium containing 1 µg·mL−1 insulin, 50 nM T3, and 0.5 µM troglitazone once every two days (three times, Days 2–6). On Day 2, BRWE was prepared in a maintenance medium at concentration of 5 and 10 µg·mL^−1^. For full differentiation of white adipocytes, 3T3-L1 cells were cultured and differentiated without troglitazone.

### 2.4. Cell Cytotoxicity Assay

The cell cytotoxicity was measured with a 3-(4,5-dimethylthiazol-2-*yl*)-5-(3-carboxymethoxyphenyl)-2-(4-sulfophenyl)-2H-tetrazolium (MTS) kit (Promega, Madison, WI, USA) as previously described [13].

### 2.5. Oil Red O staining

Intracellular lipid accumulation was measured using Oil Red-O, as described previously [12].

### 2.6. RNA Isolation and Real-Time Reverse Transcription—Polymerase Chain Reaction (RT-PCR)

RNA isolation and real-time RT-PCR were performed as previously described [14]. Briefly, the total RNA was obtained using GeneAll^R^ RiboEX Total RNA extraction (GeneAll Biotechnology, Seoul, Korea). The relative gene expressions were calculated based on the comparative CT method using the StepOne software v2.1 (Applied Biosystems, Foster City, CA, USA). The mRNA expression of *Gapdh* was used as an endogenous control. The primers used in this study are as follows: *Ucp1* (F: 5′-AACTGTACAGCGGTCTGCCT-3′, R: 5′-TAAGCCGGCTGAGATCTTGT-3′), *Pgc1a* (F: 5′-AATGCAGCGGTCTTAGCACT-3′, R: 5′-TGTTGACAAATGCTCTTCGC-3′), and *Gapdh* (F: 5′-AACTTTGGCATTGTGGAAGG-3′, R: 5′-GGATGCAGGGATGATGTTCT-3′).

### 2.7. Animal Experiments

Male C57BL/6J mice (7-week-old) were purchased from Deahan Biolink Co. (Eumsung, Korea) and kept for 1 week prior to the experiments. The mice were orally administrated BRWE (100 mg·kg^−1^, daily) or an equivalent volume of vehicle (5% DMSO in phosphate buffered-saline (PBS)) for 2 weeks. The mice were kept at 4 °C, and their rectal temperature was measured at the indicated time points. The mice were sacrificed by cervical dislocation under CO_2_ asphyxiation. The animal experiments regarding mice were performed according to a protocol approved by the Animal Care and Use Committee of the Institutional Review Board of Kyung Hee University (confirmation number: KHUASP (SE)-13-012).

Zebrafish were raised according to standard protocol [15]. Larvae obtained from the adult zebrafish crosses were raised in a 28.5 °C incubator and fed regular diets starting at 5.5 days post fertilization (dpf). BRWE at 100 ug·mL^−1^ was treated and replaced daily in fish water for 8 days starting from 10 dpf. For adipocyte visualization, the larvae were incubated for 30 min in dark in Nile red (Invitrogen) prepared from a stock solution (1.25 mg·mL^−1^ in acetone) by 1:2500 dilution. After rinsing with fish water several times, the larvae were viewed using a Leica M165FC microscope equipped with Leica DFC500. The captured fluorescent signals of Nile red were quantified using Image J 1.51v. Student’s *t*-test was used to determine statistical significance (*p* < 0.05). The experimental protocols used in the zebrafish study were approved by the committee for Ethics in Animal Experiments of Wonkwang University (WKU15-151).

### 2.8. Hematoxylin and Eosin (H&E) Staining

WAT was washed in PBS and fixed in 10% formalin for 2 weeks. Then, the tissues were embedded in paraffin. The tissue sections were deparaffinized in xylene and rehydrated with ethanol/water and then stained with hematoxylin-eosin (H&E). Microscopic examinations were performed, and photographs were taken under a regular light microscope. The average adipose droplet size was calculated using the Image J software program (National Institute of Health, Bethesda, MD, USA).

### 2.9. Western Blot Analysis

The cells and tissues were lysed through radioimmunoprecipitation assay (RIPA) buffer (Cell Signaling Technology, Danvers, MA, USA) on ice for 30 min, and then, insoluble materials were removed by centrifugation at 13,000 rpm for 20 min at 4 °C. The lysates were resolved by sodium dodecyl sulfate (SDS)-polyacrylamaide gel electrophoresis and transferred onto a PVDF membrane. Then, the membranes were blocked in 5% skim milk and incubated with the respective primary antibody (1:1000) overnight at 4 °C followed by incubation with horseradish peroxidase (HRP)-conjugated secondary antibody (1:5000) for 1 h at room temperature. The protein signals were detected using the ECL advance kit.

### 2.10. Immunofluorescence Assay

The cells and tissues were fixed using 10% formalin and blocked with 5% BSA for 1 h. After, the cells and tissue were incubated with the indicated primary antibodies (anti-UCP1 and anti-PGC1α, 1:50 in 5% BSA) overnight at 4 °C. After washing, the cells and tissue were incubated with Alexa Flour 488- or 633-conjugated secondary antibody (1:1000), and the fluorescence was detected using an EVOS^R^ Cell Imaging systems (Thermo Scientific, Carlsbad, CA, USA).

### 2.11. Statistical Analysis

Data were expressed as mean ± SEM of independent experiments. Statistical differences were calculated by one-way ANOVA and a subsequent post hoc Tukey test unless stated otherwise. All statistical analyses were completed using SPSS statistical analysis software version 11.5 (SPPS Inc., Chicago, IL, USA). All probability values (^#^ *p* < 0.05, * *p* < 0.05, ** *p* < 0.01, and *** *p* < 0.001,) were used as the criterion for statistical significance.

## 3. Results

### 3.1. Effect of BRWE on Adipocyte Differentiation in hMSCs Cells and Zebrafish

**Figure 1:**
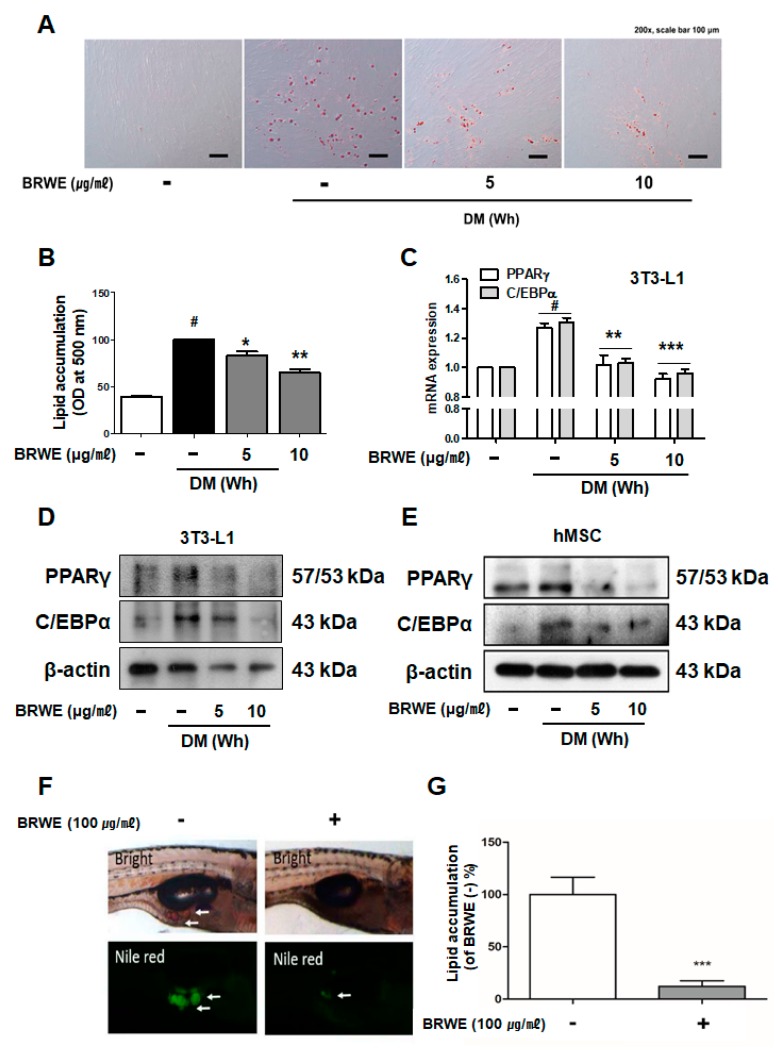
Black raspberry water extract (BRWE) suppresses adipocyte development in human adipose tissue-derived mesenchymal stem cells (hMSCs) and zebrafish. (**A**,**B**) Accumulation of intracellular lipid (lipid droplets) was measured with Oil-Red O assay in hMSCs differentiated with the presence or absence of BRWE (5 and 10 μg·mL^−1^) for 15 days. (**C**) mRNA levels of *Pparg* and *Cebpa* were measured using a Real-time RT-PCR assay. (**D**,**E**) Cell lysates were subjected to SDS-PAGE, and immunoblot analysis was performed using each antibody to PPARγ and C/EBPα. β-actin was used as a loading control. (**F**) Bright field images in the top row show both DMSO- or BRWE-treated zebrafish larvae at 17 dpf. Fluorescent adipocytes stained with Nile red are shown in the bottom row. Larvae are shown in lateral views with rostral to the right, dorsal to the top. Adipocytes are marked by white arrows. (**G**) Florescent intensities in Figure 1F were quantified using the ImageJ software and presented as a graph in arbitrary units. All data are expressed as the mean ± SEM. (Figure 1B,C) ^#^ *p* < 0.05, significantly different from DM (Wh)-untreated pre-adipocytes; * *p* < 0.05, ** *p* < 0.01, and *** *p* < 0.001, significantly different from DM (Wh)-stimulated adipocytes. (**G**) Unpaired *t*-test was used to analyze different versus the phosphate buffered-saline (PBS) group, and statistical significance was set as * *p* < 0.05. BRWE, black raspberry water extract.

Cytotoxicity of BRWE in hMSCs was confirmed in our previous report [11]; thus, we chose the concentrations of 5 and 10 μg·mL^−1^ for the treatment of the pre-adipocytes. To determine the anti-adipogenic effect of BRWE, we assessed the intracellular lipid accumulation during the differentiation of pre-adipocytes to adipocytes in hMSCs cells. As a result, BRWE attenuated the development of lipid droplets in a dose-dependent manner during white adipocyte differentiation (Figure 1A,B). Next, it was confirmed whether BRWE treatment potentially affects the expression of adipogenesis-related proteins such as peroxisome proliferator-activated receptor gamma (PPARγ) and CCAAT/enhancer-binding protein alpha (C/EBPα). As shown in Figure 1C, BRWE treatment suppressed the mRNA levels of *Pparg* and *Cebpa* in 3T3-L1 adipocytes. Furthermore, by immunoblotting assays, we observed a noticeable decrease of the levels of PPARγ and C/EBPα reduction in BRWE-treated white adipocytes (Figure 1D,E).

Furthermore, to investigate the role of BRWE during adipocyte development, we treated 100 *μ*g·ml^-1^ BRWE or 0.1% DMSO to zebrafish larva for 8 days from 10 to 17 days dpf. Zebrafish is a widely accepted experimental model for adipogenesis study because they develop rapidly, they have a short life cycle, and the genome sequence information is in detail enough for pathway investigations [16] and because lipid metabolism pathways are conserved between mammals and fish [17,18,19]. To analyze the amount of lipid, the larvae were stained with Nile red and photographed. In addition, the length of each larva was measured, since adipocyte development is known to be proportional to the size of larvae [20]. The average length of larvae did not differ significantly between the BRWE-treated group and the vehicle-treated group (5.98 and 6.03 mm in the BRWE-treated or control groups, respectively), and the range of larvae was from 5.5 to 6.5 mm. However, signal intensity of lipid visualized by Nile red displayed more than 8 times reduction in the BRWE-fed group when compared to the control group (Figure 1C,D). This result indicated a repressive effect of BRWE on white adipocyte development in zebrafish. Taken together, the treatment of BRWE had a significant suppressive impact on lipid accumulation during adipocyte development.

### 3.2. Effect of BRWE on Inguinal WAT (iWAT) Browning in Cold-Exposed Mice

**Figure 2:**
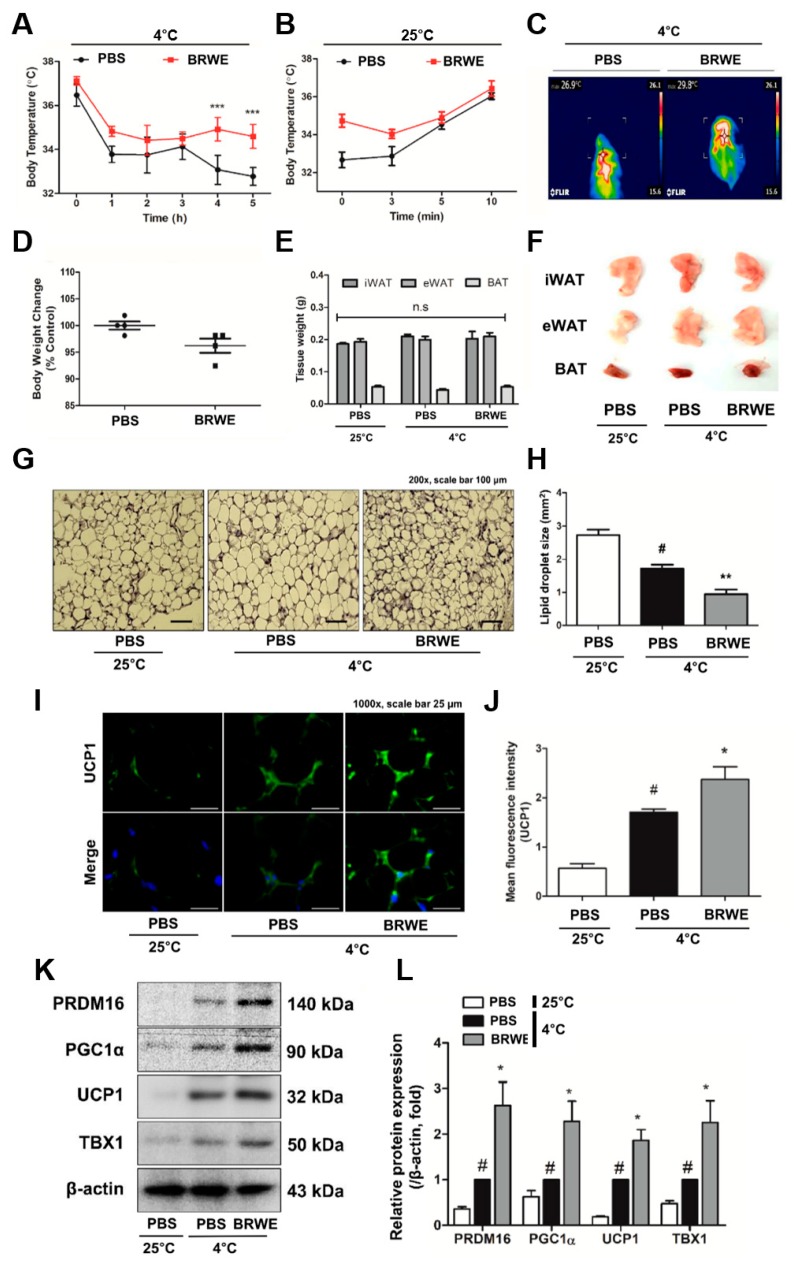
BRWE increases browning of inguinal white adipose tissue in cold-stressed C57BL/6J mice. C57BL/6J mice were treated with BRWE (100 mg·kg^−1^, oral administration) or phosphate buffered-saline (PBS) for 2 weeks and then placed at 4 °C for 5 h; (**A**,**B**) rectal temperature was measured after cold exposure: (**C**) representative infrared thermal image. (**D**) Body weight change and (**E**) adipose tissue weights were measured: (**F**) representative photograph of adipose tissues after cold exposure for 5 h. (**G**) H&E staining of iWAT (200× magnification, scale bar = 100 µm) was performed and (**H**) lipid droplet size were measured. (**I**,**J**) Immuno-fluorescent staining of iWAT (1000× magnification, scale bar = 25 µm) with anti-UCP1 antibody (green) was performed. (**K**,**L**) Western blot analysis of thermogenesis- and beige-related factors including PRDM16, PGC1α, UCP1, and TBX1 were performed. β-actin was used as a loading control. All data are expressed as the mean ± SEM. ^#^ *p* < 0.05, significantly different from blank group; * *p* < 0.05, ** *p* < 0.01, and *** *p* < 0.001, significantly different from control group. Each experiment was repeated at least 3 times, and similar results were obtained. BRWE, black raspberry water extract; iWAT, inguinal white adipose tissue.

Cold stimulation is widely known to affect remodeling of adipose tissues, i.e., BAT activation and WAT browning [21]. To confirm the effect of BRWE on the capacity of cold tolerance, we treated C57BL/6J with BRWE (100 mg·kg^−1^) by oral gavage for 12 days and conducted a cold stress test at 4 °C for 5 h. After 5 h of acute cold exposure, the rectal temperature of PBS-fed mice declined from 32.77 ± 0.41 °C and their body temperature returned to a normal state (36–37 °C) at room temperature (RT, 25 °C) after 10 min. In the BRWE-fed mice, temperature decline was significantly improved as it maintained 34.4 ± 0.54 °C of rectal temperature after 5 h, and the recuperative power of body temperature also improved compared to the PBS-fed mice (Figure 2A–C). However, body weight change and adipose tissue weight in mice with or without BRWE treatment showed no significant difference (Figure 2D–F). The results also showed that BRWE treatment affected iWAT browning. The H&E staining and bar graph shown in Figure 2G,H shows the reduced lipid droplet size in iWAT. Immunofluorescence staining was performed in iWAT of BRWE-fed or PBS-fed mice, and we could verify an extensively strong signaling of the UCP1 (green dye) displayed in BRWE-treated mice compared to the PBS-fed mice (Figure 2I,J). The beige-specific markers such as PR domain zinc-finger protein 16 (PRDM16), PGC1α, UCP1, and TBX1 were increased in iWAT of BRWE-fed and cold-exposed mice (Figure 2K,L). Furthermore, we observed the thermogenic alteration of BRWE in other adipose tissues (epididymal WAT and BAT). BRWE activated the UCP1-related thermogenic signaling in BAT while it had no effect on UCP1 expression in epididymal WAT of cold-exposed mice (Supplementary Figure S1A,B).

### 3.3. Effect of BRWE on Beige Adipocyte-Specific Markers in 3T3-L1 Adipocytes

**Figure 3:**
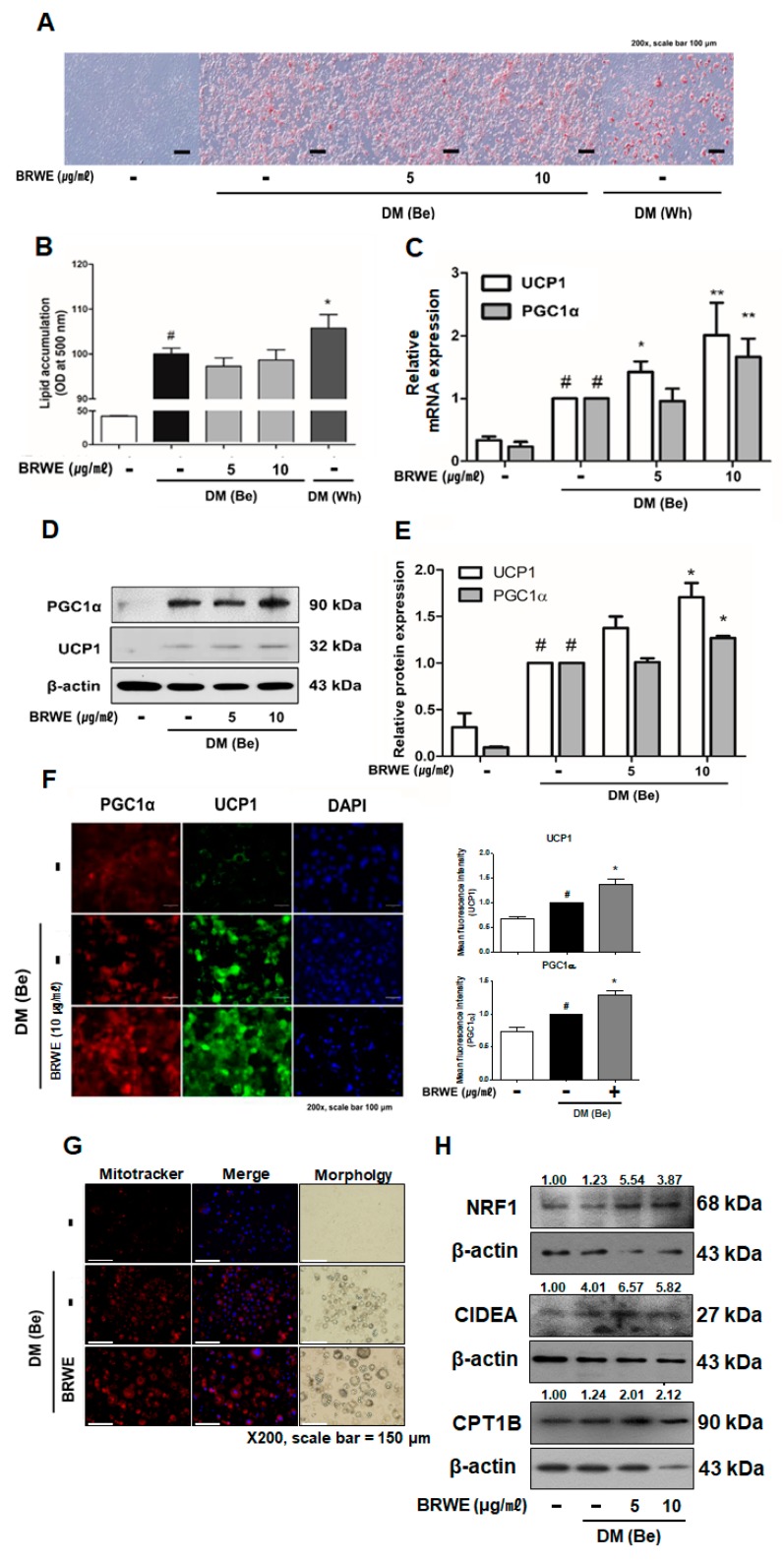
BRWE promotes browning of adipocytes in 3T3-L1 adipocytes. Two days after achieving confluence, 3T3-L1 pre-adipocyte cells were stimulated by DM (Be) containing 0.5 mM IBMX, 167 μM insulin, T3 20 μM, and 0.5 μM troglitazone with/without various concentrations of BRWE. After 6 days, intracellular lipid droplets were measured with (**A**,**B**) Oil-Red O assay and the absorbance was measured at 500 nm. (**C**) The mRNA and (**D**,**E**) protein levels of thermogenesis-related factors in 3T3-L1 with BRWE were measured. (**F**) Expression of intracellular UCP1 and PGC1α in 3T3-L1 were evaluated by immunofluorescence staining. The changes were observed at 200× (scale bar = 100 μM). Florescent intensities were quantified using the ImageJ software and presented as a graph in arbitrary units. (**G**) Mitotracker staining was performed and observed at 200× (scale bar = 150 μM). (**H**) Protein levels of NRF1, CIDEA, and CPT1B were measured. All data are expressed as the mean ± SEM. ^#^ *p* < 0.05, significantly different from DM (Be)-untreated pre-adipocytes; * *p* < 0.05 and ** *p* < 0.01, significantly different from DM (Be)-stimulated adipocytes cells. Each experiment was repeated at least 3 times, and similar results were obtained. BRWE, black raspberry water extract.

To study the efficacy of BRWE on beige induction in 3T3-L1 murine pre-adipocytes, we attempted to measure the intracellular lipid accumulation in DM (Be)-stimulated 3T3-L1 cells. The treatment with BRWE (5 and 10 μg·mL^−1^) did not affect accumulation of intracellular lipid in DM (Be)-stimulated 3T3-L1 cells (Figure 3A,B); however, we found out that mRNA expressions of thermogenic factors such as *Ucp1* and *Pgc1a* were markedly upregulated by BRWE (Figure 3C–E). In accordance with the real-time RT-PCR results, we further confirmed that the BRWE treatment also increased the expression of beige-adipocyte related proteins by using western blot and immunofluorescence staining. BRWE treatment increased the protein expression of beige markers (UCP1 and PGC1α) compared to the DM (Be) only control group (Figure 3F).

Browning within WAT can occur due to new preadipocytes that differentiate into beige adipocytes or result from trans-differentiation of mature white adipocytes into beige adipocytes [22]. Above, we could conclude BRWE can induce preadipocytes to differentiate into beige adipocytes. Thus, we next attempted to find out whether BRWE has the capability to induce browning of mature white adipocytes. Accordingly, BRWE treatment with 48 h increased the UCP1 levels in mature 3T3-L1 adipocytes of which the level was similar to the effect of the β3 AR agonist CL316,243 (Supplementary Figure S2).

Although non-shivering thermogenesis is mediated by UCP1, activation of UCP1 may not necessarily mean equal to the induction of mitochondrial thermogenesis program [23]. Thus, we measured further factors related in mitochondrial biogenesis and activation. As shown in Figure 3G, a Mitotracker staining assay revealed increased abundancy of mitochondria in BRWE-treated cells. Increased factors of mitochondrial abundance and action including cell death activator (CIDEA) [24] and nuclear respiratory factor 1 (NRF1) [25] showed the increased mitochondrial biogenesis/activation induced by BRWE, and furthermore, the increased mitochondria enzyme carnitine palmitoyltransferase 1B (CPT1B) [26] indicated increased mitochondrial β-oxidation by BRWE treatment (Figure 3H).

### 3.4. Effect of Phytochemicals Derived from BRWE on Beige Markers in 3T3-L1 Cells

**Figure 4:**
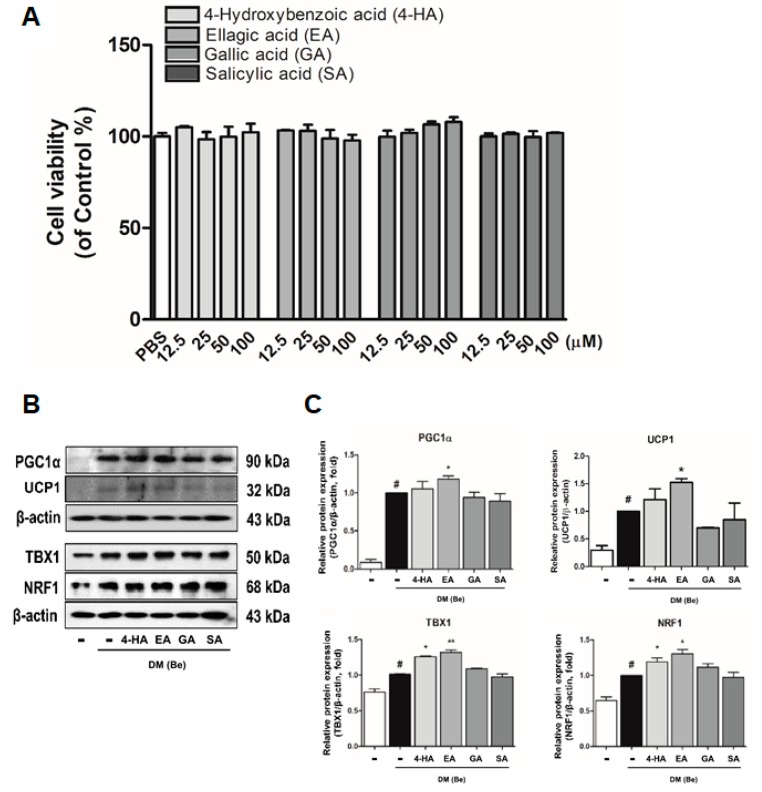
Compounds from BRWE induces browning of adipocytes in 3T3-L1 cells. (**A**) Cytotoxicity of compounds (4-hydroxybenzoic acid (4-HA), ellagic acid (EA), gallic acid (GA), and salicylic acid (SA)) in undifferentiated 3T3-L1 pre-adipocytes were measured by 3-(4,5-dimethylthiazol-2-*yl*)-5-(3-carboxymethoxyphenyl)-2-(4-sulfophenyl)-2H-tetrazolium (MTS) assay, and the absorbance was measured at 490 nm. (**B**,**C**) The protein levels of thermogenesis- and beige-related factors including UCP1, PGC1α, TBX1, and NRF1 were evaluated in 3T3-L1 cells treated with BRWE. β-actin was used as a loading control. All data are expressed as the mean ± SEM. ^#^ *p* < 0.05, significantly different from DM (Be)-untreated pre-adipocytes; * *p* < 0.05 and ** *p* < 0.01, significantly different from DM (Be)-stimulated adipocytes cells. Each experiment was repeated at least 3 times, and similar results were obtained. BRWE, black raspberry water extract.

In our previous study, we confirmed the phytochemical profile of BRWE using HPLC MS/MS analysis which identified four major compounds (4-hydroxybenzoic acid (4-HA), ellagic acid (EA), gallic acid (GA), and salicylic acid (SA)) [12]. Treatment of aforementioned compounds in vitro were carried out to evaluate their potential effects on the expression of beige-specific markers. First, the cytotoxicity of these constituents was measured. Murine 3T3-L1 pre-adipocytes were treated with various concentrations (12.5 to 100 µM) of the four major compounds for 48 h. As shown in Figure 4A, the cell viability was not affected up to the concentration of 100 µM of all compounds. We next investigated whether four compounds treatment could affect beige markers (PGC1α, UCP1, TBX1, and NRF1) in DM (Be)-stimulated 3T3-L1 cells. Interestingly, the results showed that EA-treated 3T3-L1 cells significantly increased the beige markers when compared against DM (Be)-stimulated 3T3-L1 cells (Figure 4B,C), while the other three constituents (4-HA, GA, and SA) failed to display such an effect.

## 4. Discussion

Obesity is an abnormal condition of immoderate lipid accumulation in the body, and this stage accelerates a pathogenic potential growth in further chronic diseases [27]. Thus, developing new drugs to combat obesity is still an ongoing challenge, and interest on natural products which display fewer side effects than artificial medicines such as headache, nausea, and dizziness is rapidly growing [28].

PPARγ and C/EBPα play major transcription factors in adipocyte differentiation and function [29]. Activation of these factors promotes lipid accumulation by inducing adipogenic- and lipogenic-related factors such as sterol regulatory element binding protein-1c (SREBP-1c) or adipocyte fatty acid-binding protein (aP2) [30,31]; thus, regulation of PPARγ and C/EBPα can possibly lead to the reduction of excessive lipid accumulation. Previous studies demonstrated that BR is a potential candidate for treating obesity via a negative regulation of PPARγ and C/EBPα in 3T3-L1 adipocytes [11]. Herein, we confirmed that the BRWE significantly inhibits lipid accumulation in hMSC adipocytes as well by decreasing the expression of PPARγ and C/EBPα (Figure 1A,B) and also reduces lipid development in zebrafish (Figure 1C,D). Therefore, this study provides supplementary evidence in support of the anti-obesity effect of BRWE.

Thermogenesis in the adipose tissue plays an essential role in the whole-body energy homeostasis, thereby being considered for a new therapeutic target for obesity treatment [32]. In this phenomenon, UCP1 acts as a vital element as it converts the idling of the mitochondrial electron transport chain. This results in the release of protons and thus leads to generation of heat instead of synthesis of adenosine tri-phosphate (ATP) [33]. The brown/beige adipocytes are known as the UCP1-positive adipocytes which possess thermogenic capacity in response to cold exposure or β3 AR activation [34]. In addition, in response to cold stress, the activation of PRDM16, a key regulator of beige differentiation, lead to transcriptive activation of beige specific genes such as *Cebpb*, *Pgc1a*, *Tbx1*, and *Nrf1* [35,36,37]. These factors hydrolyze triacylglycerol (TG) into FFAs via the activation of intracellular lipases including adipose triglyceride lipase (ATGL), hormone-sensitive lipase (HSL), and monoacylglycerol lipase (MGL) and eventually induce mitochondrial biogenesis and activation [38,39]. The released FFAs serve as fuel of adaptive thermogenesis [40,41]. The present study confirmed that BRWE treatment decreased lipid droplet size in iWAT and also promoted browning by increasing the expressions of PRDM16, PGC1α, TBX1, and UCP1 in cold-exposed mice (Figure 2). Furthermore, we determined that BRWE increased both mRNA expressions and protein levels of PGC1α and UCP1 in 3T3-L1 cells. Based on our findings, it is believed that BRWE can promote beige induction in iWAT of cold-exposed mice and 3T3-L1 cells (Figure 3).

It has been reported that numerous phenolic compounds display strong anti-obesity properties in vitro and in vivo models. Especially, recent studies have reported the browning effect of such nature-derived materials. Briefly, trans-cinnanic acid (*t*CA), isolated from *Cinnamomum cassia*, was reported to induce browning in white adipocytes via activating the β3 AR, UCP1, and AMPK signaling pathway in 3T3-L1 cells [42], and genistein, a component of *Glycine max*, can promote induction of WAT browning by increasing PRDM16, UCP1, and PGC1α in C57BL/6 mice [43]. In order to confirm the effect of phytochemicals which compose BRWE on beige differentiation, we performed further experiments. As our previous study indicated that BRWE contains various bioactive compounds including 4-HA, EA, GA, and SA [12], we evaluated the effect of these four constituents. As a result, EA, one of the major phytochemicals in BRWE, induced beige adipocyte differentiation/activation by increasing UCP1, PGC1α, TBX1, and NRF1 expressions (Figure 4).

## 5. Conclusions

This study clarified the anti-obesity effect of BRWE and demonstrated three important findings. First, the treatment of BRWE inhibited lipid accumulation in hMSCs and zebrafish. Second, BRWE activated beige differentiation by inducing UCP1/PGC1α expression in 3T3-L1 adipocytes and increased iWAT browning in vivo as confirmed by increased beige markers (PRDM16, UCP1, PGC1α, and TBX1) in cold-exposed mice. Last, EA, one of the phytochemicals composing BRWE, promoted beige induction in 3T3-L1 adipocytes.

By our previous studies on the effect of BRWE [11,12], we have shown BRWE can suppress lipid accumulation in WAT and induce thermogenesis in BAT. In the current report, we observed that BRWE can induce browning of white adipocytes to functionally display UCP1-mediated thermogenesis as well. Taken together, our findings suggest that BRWE can promote beige differentiation in vitro and in vivo and thus is a potential agent for obesity care; however, further investigations are necessary to determine the exact pathways of beige differentiation by EA.

## Supplementary Materials

The following are available online at https://www.mdpi.com/2072-6643/11/9/2164/s1, Figure S1: Effect of BRWE on epididymal WAT browning and BAT activation in cold-exposed mice; Figure S2: Effect of BRWE on trans-differentiation of mature white adipocytes into beige adipocytes.

## References

[1] El-shiekh R.A., Al-Mahdy D.A., Mouneir S.M., Hifnawy M.S., Abdel-Sattar E.A. (2019). Anti-obesity effect of argel (Solenostemma argel) on obese rats fed a high fat diet. J. Ethnopharmacol..

[2] Kopelman P.G. (2000). Obesity as a medical problem. Nature.

[3] Gu X., Hong Y., Lin Y., Si Q., Yang L., Dong W. (2019). Ginsenoside Rb2 alleviates obesity by activation of brown fat and induction of browning of white fat. Front. Endocrinol..

[4] Shabalina I.G., Petrovic N., de Jong J.M., Kalinovich A.V., Cannon B., Nedergaard J. (2013). UCP1 in brite/beige adipose tissue mitochondria is functionally thermogenic. Cell Rep..

[5] Chou Y.-C., Ho C.-T., Pan M.-H. (2018). Immature Citrus reticulata extract promotes browning of beige adipocytes in high-fat diet-induced C57BL/6 mice. J. Agric. Food Chem..

[6] Lo K.A., Sun L. (2013). Turning WAT into BAT: A review on regulators controlling the browning of white adipocytes. Biosci. Rep..

[7] Wang W., Seale P. (2016). Control of brown and beige fat development. Nat. Rev. Mol. Cell Biol..

[8] Nedergaard J., Golozoubova V., Matthias A., Asadi A., Jacobsson A., Cannon B. (2001). UCP1: The only protein able to mediate adaptive non-shivering thermogenesis and metabolic inefficiency. Biochim. Biophys. Acta-Bioenerg..

[9] Concha F., Prado G., Quezada J., Ramirez A., Bravo N., Flores C., Herrera J., Lopez N., Uribe D., Duarte-Silva L. (2019). Nutritional and non-nutritional agents that stimulate white adipose tissue browning. Rev. Endocr. Metab. Disord..

[10] Lim J.W., Jeong J.T., Shin C.S. (2012). Component analysis and sensory evaluation of Korean black raspberry (Rubus coreanus Mique) wines. Int. J. Food Sci. Technol..

[11] Jeong M.-Y., Kim H.-L., Park J., An H.-J., Kim S.-H., Kim S.-J., So H.-S., Park R., Um J.-Y., Hong S.-H. (2013). Rubi fructus (Rubus coreanus) inhibits differentiation to adipocytes in 3T3-L1 cells. Evid.-Based Complement Altern. Med..

[12] Jeong M., Kim H., Park J., Jung Y., Youn D., Lee J., Jin J., So H., Park R., Kim S. (2015). Rubi Fructus (Rubus coreanus) activates the expression of thermogenic genes in vivo and in vitro. Int. J. Obes..

[13] Kim H.-L., Park J., Park H., Jung Y., Youn D.-H., Kang J., Jeong M.-Y., Um J.-Y. (2015). Platycodon grandiflorum A. de candolle ethanolic extract inhibits adipogenic regulators in 3T3-L1 cells and induces mitochondrial biogenesis in primary brown preadipocytes. J. Agric. Food Chem..

[14] Kim H.-L., Park J., Jung Y., Ahn K.S., Um J.-Y. (2019). Platycodin D, a novel activator of AMP-activated protein kinase, attenuates obesity in db/db mice via regulation of adipogenesis and thermogenesis. Phytomedicine.

[15] Kimmel C.B., Ballard W.W., Kimmel S.R., Ullmann B., Schilling T.F. (1995). Stages of embryonic development of the zebrafish. Dev. Dyn..

[16] Ablain J., Zon L.I. (2013). Of fish and men: Using zebrafish to fight human diseases. Trends Cell Biol..

[17] MacDonald M.E., Stainier D.Y.R. (2007). Lessons from “Lower” Organisms: What Worms, Flies, and Zebrafish Can Teach Us about Human Energy Metabolism. PLoS Genet..

[18] Oka T., Nishimura Y., Zang L., Hirano M., Shimada Y., Wang Z., Umemoto N., Kuroyanagi J., Nishimura N., Tanaka T. (2010). Diet-induced obesity in zebrafish shares common pathophysiological pathways with mammalian obesity. BMC Physiol..

[19] Anderson J.L., Carten J.D., Farber S.A. (2011). Zebrafish lipid metabolism: From mediating early patterning to the metabolism of dietary fat and cholesterol. Methods Cell Biol..

[20] Imrie D., Sadler K.C. (2010). White adipose tissue development in zebrafish is regulated by both developmental time and fish size. Dev. Dyn..

[21] Jung Y., Park J., Kim H.-L., Sim J.-E., Youn D.-H., Kang J., Lim S., Jeong M.-Y., Yang W.M., Lee S.-G. (2017). Vanillic acid attenuates obesity via activation of the AMPK pathway and thermogenic factors in vivo and in vitro. FASEB J..

[22] Ikeda K., Maretich P., Kajimura S. (2018). The Common and Distinct Features of Brown and Beige Adipocytes. Trends Endocrinol. Metab..

[23] Chang S.-H., Song N.-J., Choi J.H., Yun U.J., Park K.W. (2019). Mechanisms underlying UCP1 dependent and independent adipocyte thermogenesis. Obes. Rev..

[24] Nishimoto Y., Tamori Y. (2017). CIDE Family-Mediated Unique Lipid Droplet Morphology in White Adipose Tissue and Brown Adipose Tissue Determines the Adipocyte Energy Metabolism. J. Atheroscler. Thromb..

[25] Bartelt A., Widenmaier S.B., Schlein C., Johann K., Goncalves R.L.S., Eguchi K., Fischer A.W., Parlakgül G., Snyder N.A., Nguyen T.B. (2018). Brown adipose tissue thermogenic adaptation requires Nrf1-mediated proteasomal activity. Nat. Med..

[26] Calderon-Dominguez M., Sebastián D., Fucho R., Weber M., Mir J.F., García-Casarrubios E., Obregón M.J., Zorzano A., Valverde Á.M., Serra D. (2016). Carnitine Palmitoyltransferase 1 Increases Lipolysis, UCP1 Protein Expression and Mitochondrial Activity in Brown Adipocytes. PLoS ONE.

[27] Fu J., Oveisi F., Gaetani S., Lin E., Piomelli D. (2005). Oleoylethanolamide, an endogenous PPAR-α agonist, lowers body weight and hyperlipidemia in obese rats. Neuropharmacology.

[28] González-Castejón M., Rodriguez-Casado A. (2011). Dietary phytochemicals and their potential effects on obesity: A review. Pharmacol. Res..

[29] Kim H.-L., Sim J.-E., Choi H.-M., Choi I.-Y., Jeong M.-Y., Park J., Jung Y., Youn D.-H., Cho J.-H., Kim J.-H. (2014). The AMPK pathway mediates an anti-adipogenic effect of fruits of Hovenia dulcis Thunb. Food Funct..

[30] Horton J.D., Goldstein J.L., Brown M.S. (2002). SREBPs: Activators of the complete program of cholesterol and fatty acid synthesis in the liver. J. Clin. Investig..

[31] Lowell B.B. (1999). An essential regulator of adipogenesis and modulator of fat cell function: PPARγ. Cell.

[32] Sell H., Deshaies Y., Richard D. (2004). The brown adipocyte: Update on its metabolic role. Int. J. Biochem. Cell Biol..

[33] Kajimura S., Spiegelman B.M., Seale P. (2015). Brown and beige fat: Physiological roles beyond heat generation. Cell Metab..

[34] Cannon B., Nedergaard J. (2004). Brown adipose tissue: Function and physiological significance. Physiol. Rev..

[35] Wu Z., Boss O. (2007). Targeting PGC-1α to control energy homeostasis. Expert Opin. Ther. Targets.

[36] Lee P., Linderman J.D., Smith S., Brychta R.J., Wang J., Idelson C., Perron R.M., Werner C.D., Phan G.Q., Kammula U.S. (2014). Irisin and FGF21 are cold-induced endocrine activators of brown fat function in humans. Cell Metab..

[37] Wu J., Boström P., Sparks L.M., Ye L., Choi J.H., Giang A.-H., Khandekar M., Virtanen K.A., Nuutila P., Schaart G. (2012). Beige adipocytes are a distinct type of thermogenic fat cell in mouse and human. Cell.

[38] Zhao J., Cannon B., Nedergaard J. (1998). Thermogenesis is β3-but not β1-adrenergically mediated in rat brown fat cells, even after cold acclimation. Am. J. Physiol.-Regul. Integr. Comp. Physiol..

[39] Frühbeck G., Méndez-Giménez L., Fernández-Formoso J.-A., Fernández S., Rodriguez A. (2014). Regulation of adipocyte lipolysis. Nutr. Res. Rev..

[40] Kajimura S., Seale P., Kubota K., Lunsford E., Frangioni J.V., Gygi S.P., Spiegelman B.M. (2009). Initiation of myoblast to brown fat switch by a PRDM16–C/EBP-β transcriptional complex. Nature.

[41] Matsukawa T., Villareal M.O., Motojima H., Isoda H. (2017). Increasing cAMP levels of preadipocytes by cyanidin-3-glucoside treatment induces the formation of beige phenotypes in 3T3-L1 adipocytes. J. Nutr. Biochem..

[42] Kang N.H., Mukherjee S., Yun J.W. (2019). Trans-Cinnamic Acid Stimulates White Fat Browning and Activates Brown Adipocytes. Nutrients.

[43] Palacios-González B., Vargas-Castillo A., Velázquez-Villegas L.A., Vasquez-Reyes S., López P., Noriega L.G., Aleman G., Tovar-Palacio C., Torre-Villalvazo I., Yang L.-J. (2019). Genistein increases the thermogenic program of subcutaneous WAT and increases energy expenditure in mice. J. Nutr. Biochem..

