# Supplementary material for: Black Raspberry (Rubus coreanus Miquel) Promotes Browning of Preadipocytes and Inguinal White Adipose Tissue in Cold-Induced Mice
Source: Nutrients. 2019 Sep 10;11(9):2164. doi: 10.3390/nu11092164 (PMC6769844; doi:10.3390/nu11092164)
Supplement: Supplementary file 1 [file nutrients-11-02164-s001.pdf]

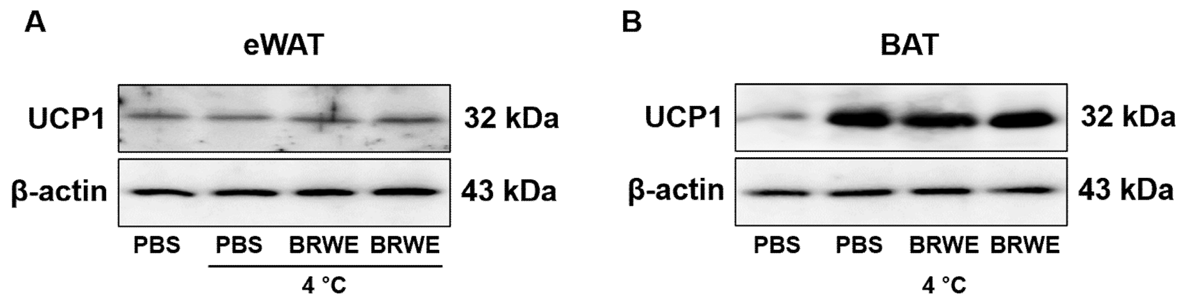

Supplementary Figure S1. Effect of BRWE on epididymal WAT browning and BAT activation in cold-exposed mice. C57BL/6J mice were treated with BRWE (100 mg·kg^{-1}, oral administration) or phosphate buffered-saline (PBS) for 2 weeks, and then placed at 4°C for 5 h. Western blot analysis of UCP1 were performed in (A) eWAT and (B) BAT. β-actin was used as a loading control. BRWE, black raspberry water extract; eWAT, epididymal white adipose tissue; BAT, brown adipose tissue.

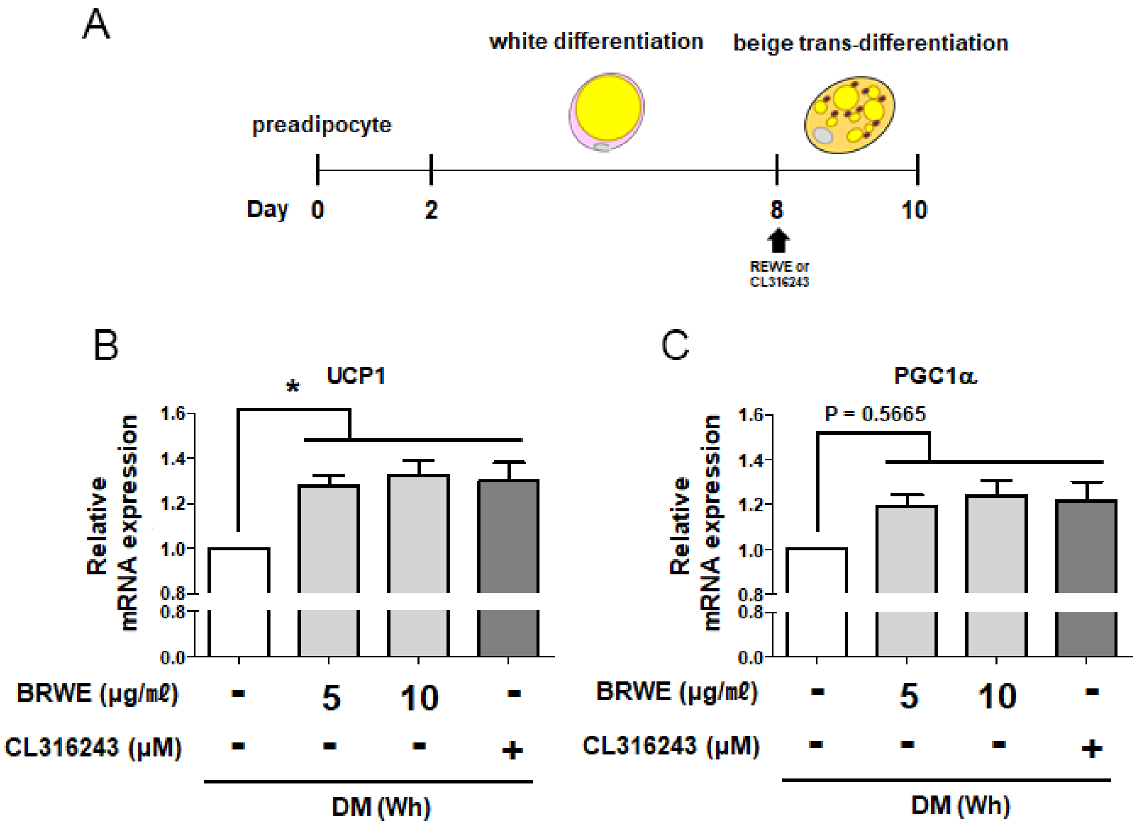

Supplementary Figure S2. Effect of BRWE on trans-differentiation of mature white adipocytes into beige adipocytes. (A) Experimental scheme to evaluate the effect on beige trans-differentiation in 3T3-L1 mature adipocytes. Real-time RT-PCR analysis of (B) *Ucp1* and (C) *Pgc1a* were performed. All data are expressed as the mean ± SEM. $^{*}p < 0.05$, significantly different from vehicle-treated adipocytes. Each experiment was repeated 3 times, and similar results were obtained. BRWE, black raspberry water extract.
